# Consumers in the Circular Economy: A Path Analysis of the Underlying Factors of Purchasing Behaviour

**DOI:** 10.3390/ijerph191811333

**Published:** 2022-09-09

**Authors:** Andrea Szilagyi, Lucian-Ionel Cioca, Laura Bacali, Elena-Simina Lakatos, Andreea-Loredana Birgovan

**Affiliations:** 1 Faculty of Industrial Engineering, Robotics and Product Management, Technical University of Cluj-Napoca, 400641 Cluj-Napoca, Romania; 2 Institute for Research in Circular Economy and Environment Ernest Lupan, 400609 Cluj-Napoca, Romania; 3 Faculty of Engineering, Lucian Blaga University of Sibiu, 550024 Sibiu, Romania

**Keywords:** circular economy, consumers, purchase behaviour, sustainability, path analysis

## Abstract

One of the major obstacles to the adoption of the circular economy is the lack of consumer interest and awareness. Despite this, the unique role of consumers in the circular economy is an understudied topic, as the literature tends to focus rather on the application of circular practices in the organizational and industrial sectors. This paper aims to examine the individual-level factors that have an influence over circular purchasing behavior. Specifically, this paper elaborates an explicative path model of purchasing circular products that takes into account environmental concern, climate skepticism, and the attitudinal factor towards circular products. The final sample consisted of 566 respondents from Romania. Our findings showed that environmental concern has a positive significant impact on circular purchase behaviour and this relationship is mediated by the attitude towards circular products. Moreover, those with high levels of climate skepticism showed an increased level of perceived greenwashing among organizations.

## 1. Introduction

The transition to a circular economy is a critical step toward achieving the sustainable development goals. Individuals, organizations, and the scientific community have begun to advocate for a shift away from a linear, reductive approach to economic growth and toward a holistic, sustainable approach that closes the resource consumption loop [1,2,3]. However, in order to understand and support the transition to a circular economy, people must be equipped with appropriate thinking and concepts. Understanding and involving consumers is crucial during this transition in order to shape their behaviours toward sustainable consumption habits [4]. As empirical evidence suggests, one of the major obstacles to the adoption of the circular economy is a lack of consumer interest and awareness [5]. However, even so, acknowledging one’s own contribution is not enough, as effective action with visible outcomes in real life is required to combat today’s profligate lifestyle habits.

Despite this, the unique role of consumers in the circular economy is, however, an under researched subject as the literature tends to focus rather on the application of circular practices in the organizational and industrial sectors. Nevertheless, it is also true that consumers exhibit volatility within the ecological market, which makes them a complex subject to study and analyse. On the other side, in practice, marketing experts are actively seeking to comprehend consumer demand and trends for every new circular product that they aim to launch.

The motivations behind consumer choices are multiple and can vary over time and circumstances. However, in order to realize the maximum potential of the circular economy’s inner loops (recycle–reuse–reduce), consumers’ perceptions and behaviors toward the circular products and services involved must change [6,7,8].

Green or eco-friendly products, regardless of market, are designed with the goal of conserving natural resources; for example, by incorporating recycled content or the recycling process itself into the manufacturing process [9]. The concept of a product’s circularity relates to the extent to which the materials it is made of (and their value) enable the product the potential to preserve itself over time and circulate in the economy, producing as little or no waste when its life cycle is over [10].

Thus, the primary goal of this research is to examine the individual-level factors that have an influence over circular purchasing behavior. One of the conclusions of a systematic review on consumption in the circular economy [11], which looked at a sample of 111 papers, was that less research has been done on how to boost change at the individual and collective levels to support in the spread of circular solutions and the shift to a circular economy. According to previous research [12], some of the important factors in triggering change at the individual level related to purchasing eco-friendly products are the individual’s environmental values and knowledge, which can be reflected in their level of environmental concern or climate skepticism, the attitude toward the expected outcome, and the trust in the agent or the organization offering the respective product. Therefore, this paper elaborates on an explicative path model of purchasing circular products that takes into account environmental concern, climate skepticism, and the attitudinal factor towards circular products. Moreover, the impact of climate skeptcisim on the perceived greewashing in which organizations can be engaged is investigated.

This paper continues with Section 2, dedicated to the state-of-the-art of the literature and the hypothesis development. The methodology of this study, including sample and data collection, measurement instruments, and data processing, is described in Section 3. Section 4 presents the results obtained following the path analysis, including the overall model fit and the regression weights for each studied path. Discussions regarding the relevance of the findings, limits, and recommendations for additional research are included in Section 5. Finally, Section 6 ends with a summary of the findings obtained. 

## 2. Literature and Hypothesis Development

### 2.1. Environmental Concern and Circular Products

The concern for sustainable consumption has grown among consumers over the last three decades. The explanation is rather intuitive; that is, as customers became more conscious of their role in the environmental issues caused by overconsumption, they started to purchase more eco-friendly products [13,14]. While fulfilling human consumption needs continues to be at the heart of consumer behavior, environmental preservation has also increased in importance [15,16]. As a result, a series of studies interested in the predictive factors at the individual level of ecological products have emerged. Some authors have highlighted a variance in green product purchase behavior that can also be explained by socio-demographic characteristics such as a high level of education, above-average incomes, and the specific age of the so-called millennial generation [17,18]. In the most significant theoretical frameworks used to predict ecological consumer behavior, the concept of environmental concern is prominently featured [19,20]. This variable can be conceptualized as individuals’ concern for reducing pollution and the degradation of natural resources [21,22]. Moreover, concern for the environment is one of the beliefs that constitute the attitude within the theory of planned behavior [17]. As confirmed by Newton et al. [21], concerned consumers are more likely to make informed decisions about the products they buy. For instance, customers who examine food labels before making purchases and think that these items may have benefits for their health or the environment are more likely to express a favorable purchase intention for waste-to-value food [22]. In this way, the action of seeking information can increase the likelihood of making environmentally conscious choices related to the purchase of circular products. On the other hand, actively seeking information can result in a growing susceptibility of the consumer towards a company’s transparency regarding the environmental aspects of its products. In this paper, greenwashing is defined as the deliberate distortion of a company’s environmental practices and impact on consumers [23,24].

**Hypothesis** **1a.** *Environmental concern has a positive significant impact on circular purchasing behaviour*.

**Hypothesis** **1b.** *Environmental concern has a positive significant impact on perceived greenwashing*.

### 2.2. Attitudes towards Circular Products and Purchasing Behaviour

Past research demonstrated that understanding a consumer’s environmental attitudes and behavior is a challenging, but crucial notion for addressing the profile of the environmentally conscious consumer [25]. However, a lack of product category specificity, such as circular products, and the failure to discuss or compare particular types of ecological products are two aspects that have been omitted [26,27].

The relationship between attitude and behaviour has already been examined by Ajzen and Fishbein within the theory of planned behaviour [28,29,30]. This theory is founded on the idea that our intentions are a result of our beliefs. Some of these beliefs have an impact on a person’s attitude toward the behavior. This attitude toward performing a specific behavior is specifically related to the beliefs that performing the behavior will result in specific outcomes. Thus, consumers who have more positive beliefs about circular products, supported by their environmental concern, will be more likely to engage in purchase behaviour [31]. Moreover, there is evidence of the existence of a type of customer who is more inclined to make personal efforts for the cause of a circular economy transition and is less bound to financial compensation as a motivator to participate [32]. In the same direction, in Naingolan and collaborators’ research [33], the attitudinal factors were discovered to be statistically significant in explaining household preferences for alternative waste sorting schemes. Furthermore, the idea of planned behavior models has been shown to be helpful in describing and predicting purchasing behavior for organic goods, according to Sparks and Shepherd [34].

**Hypothesis** **2a.** *The positive attitude towards circular products has a positive significant impact on circular purchasing behaviour*.

**Hypothesis** **2b.** *The positive attitude towards circular products mediates the relationship between environmental concern and circular purchasing behaviour*.

### 2.3. Climate Skepticism and Circular Products

Although there are many elements that negatively affect the climate, scientific evidence suggests that anthropogenic activities are mostly to blame for the majority of the global warming that has been observed over the last decades [35,36]. The connection between climate change skepticism and other perceptual aspects of climate change, however, has rarely been taken into account in studies. The public’s ongoing skepticism about the trends, causes, and effects of climate change has been shown to have a significant impact on mitigation and adaptation behaviors, according to more detailed academic research [37,38]. Additionally, those who are skeptical of climate change are less likely to favor mitigation strategies like emissions trading schemes or investments in renewable energy [39]. Other empirical evidence [40] supports the idea that circular business models do not adequately manage to address the psychological, social, and cultural demands of consumers, which in turn creates barriers to the spread of circular products. Specifically, even when alternative circular products are not the subject of greenwashing, customers’ cognitive biases may cause them to have a negative perception of them. Thus, all of these previous findings suggest that a high level of climate skepticism could predispose individuals on one hand to have a negative attitude towards circular products and, on the other hand, to believe that companies are more likely to be engaged in greenwashing.

**Hypothesis** **3a.** *Climate skepticism has a positive significant impact on perceived greenwashing*.

**Hypothesis** **3b.** *Climate skepticism has a negative significant impact on attitude towards circular products*.

## 3. Materials and Methods

### 3.1. Sample and Data Collection

Using the non-probability convenience sampling method, we collected data from 580 individuals in total. The data collection took place between March and June 2022 and targeted Romanian consumers. The final sample consisted of 566 respondents, as 14 responses were discarded owing to having incomplete data. For dissemination purposes, an online survey with all of the studied variables was created and uploaded to the E-survey and Google form platforms. The authors were responsible for creating the questionnaire on the aforementioned platforms as well as responding to any concerns participants might have about their participation in the study. The questionnaire was disseminated by the authors on social platforms such as Facebook or Linked-In. Following the completion of the data collection phase in June, the authors proceeded to create one database using SPSS software and validate the responses received.

Detailed socio-demographic characteristics of the sample are provided in Table 1. All participants agreed to take part in this study and provided permission for their data to be used for research purposes. Data collection was completely voluntary and anonymous, and participants were informed that their information would be kept confidential.

### 3.2. Measurement Instruments

All of the instruments used to measure the variables were developed by adapting scales from previous studies that had already demonstrated adequate psychometric properties. Table 2 contains a detailed description of each instrument used as well as their internal reliability. Environmental concern was measured by adapting the scale used by Gam [41] and Testa et al. [19]. Climate skepticism was measured using the scale developed by Whitmarsh [42]. Perceived greenwashing was measured with the scale created by Leonidou and Skarmeas [43]. The attitude towards circular products was measured by adapting the instrument used by Chen and Chai [9]. Participants were asked to rate how they perceive repaired products and products containing recycled or reused components by four essential criteria: life cycle duration, design, performance, and quality of materials. Finally, circular purchasing behaviour was measured by adapting the scale created by Testa et al. [19] and Lee [44].

All instruments demonstrated adequate internal consistency, exceeding the 0.7 agreed threshold [45]. 

### 3.3. Data Analysis

In order to test the proposed hypothesis, we used the structural equation modeling (SEM) approach. The collected data were analyzed using AMOS v.25 and IBM SPSS Statistics for Windows v.25 softwares (IBM Corp., Armonk, NY, USA). Parameter estimation for the model was performed with the maximum-likelihood parameter, as we eliminated the missing data cases. We chose the path analysis because it is an effective technique for modeling the causal relationships between several variables at once, including mediators as well.

First, preliminary and exploratory data analyses were carried out to find any potential flaws in the data as well as any missing data cases.

Descriptive statistics were computed for each variable including means, standard deviations, and correlations. All variables were normally distributed, as demonstrated by the skewness and kurtosis value.

For the model fit assessment, we took into consideration the recommendation by Hu and Bentler [46] to use multiple indices for evaluating model fit: the chi-square test value (χ^2^) with the corresponding *p*-value (*p* > 0.05), the Tucker–Lewis index (TLI ≥ 0.95), the comparative fit index (CFI ≥ 0.95), the root-mean-squared error of approximation (RMSEA ≤ 0.06), and finally the standardized root mean square residual (SRMR < 0.08).

The potential direct and indirect effects of the environmental concern and climate skepticism on circular purchase behaviour were tested and the regression weights were examined.

## 4. Results

### 4.1. Preliminary Analysis

Table 3 illustrates the descriptive findings for the studied variables. The obtained results indicated moderated levels of perceived greenwashing, attitude towards circular products, and purchase behaviour. Environmental concern is the variable with the highest levels. All of the variables are normally distributed as the swekness and kurtosis are situated between the benchmark of ±2.

Table 4 also summarizes the bivariate, zero-order Pearson’s correlation coefficients between all of the study variables. As expected, we found a negative correlation between environmental concern and climate skepticism (R = −0.359, *p* < 0.001). There is also a positive significant correlation between the positive attitude towards circular products and actual purchase behaviour (R = −0.396, *p* < 0.001). Moreover, the correlation between environmental concern and purchase behaviour was significant (R = 0.270, *p* < 0.001).

### 4.2. Model Fit Assessment

Eight typically used indicators can be used to evaluate the model’s fit, including chi-square to the degree of freedom (χ^2^/df), the goodness of fit index (GFI), the adjusted goodness of fit index (AGFI), the comparative fit index (CFI), the Tucker–Lewis index (TLI), the root mean square error of approximation (RMSEA), the standardized root mean square residual (SRMR), and the normative fit index (NFI) [46,47].

The results (Table 5) provided a good fit to the data; χ^2^= 0.176, CFI = 1.00, NFI = 0.998, TLI = 1.032, RMSEA = 0.000, and SRMR = 0.007.

RMSEA indicates how well the proposed model fit with the real situation in the population. Values between 0.08 and.10 are suitable, whereas values less than 0.05 indicate a strong fit [48,49]. As Table 5 shows, the proposed model indicates a strong fit.

CFI contrasts the assumed model with a model without assumed relationships. It has an upper bound of 1 and any value exceeding 0.95 is considered to be an appropriate level of model fit [46]. The proposed model exceeded the critical threshold, thus it can be considerate a good fit.

### 4.3. Hypothesis Testing

**Figure 1:**
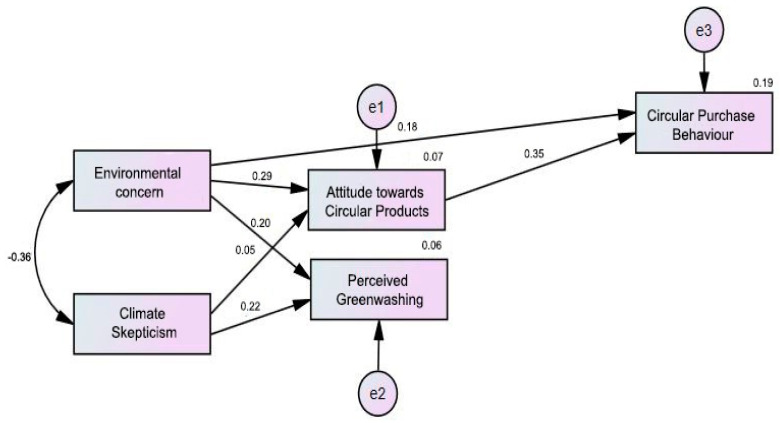
Path model with standardised parameter estimates (e1, e2, e3 = unobserved errors).

The final path model tested with AMOS software with standardised parameter estimates is presented in Figure 1. The maximum likelihood estimation was used as the final sample did not contain any missing data cases.

Our first hypothesis was that environmental concern has a significant positive impact on circular purchase behaviour. This hypothesis received empirical support (β = 0.176, SE = 0.04, *p* < 0.001), as can be seen in Table 6. Hypothesis 1b shows that, among consumers, environmental concern significantly predicts the perceived greenwashing of organizations. In other words, the regression weight for environmental concern in the prediction of perceived greenwashing is significantly different from zero at the 0.001 level.

Moreover, a positive attitude towards circular products significantly predicts circular purchase behaviour (β = 0.349, SE = 0.46, *p* < 0.001), as Hypothesis 2a stated in the beginning.

Regarding Hypothesis 2b, which proposed the significant mediating effect of the attitudinal factor, as the value of indirect and direct effects are significant, it demonstrated the existence of mediation effects of the attitude towards circular products within the relationship of environmental concern and circular purchase behaviour. Therefore, Hypothesis 2b is also accepted.

Hypothesis 3a stated that those with a higher level of climate skepticism will tend to show a high level of perceived greenwashing as well. The hypothesis received empirical support. As illustrated in Table 6, the probability of obtaining a critical ratio as large as 4.942 in absolute value is less than 0.001.

Finally, Hypothesis 3b did not receive empirical support. Thus, we rejected the hypothesis that climate skepticism has a negative impact on circular purchase behaviour (β = 0.05, SE = 0.40, *p* = 0.20.).

## 5. Discussion

### 5.1. Theoretical and Practical Implications

Regarding Hypothesis 1a, it was demonstrated that concern for protecting the environment positively predicts the purchase behavior of circular products. The result obtained is similar to that of other studies that have researched the concern for the environment and related it to similar concepts such as proactive environmental protection behaviors [50]. Moreover, a high level of environmental concern has a positive significant impact on the perceived greenwashing (Hypothesis 1b). This could be because those with a higher level of environmental concern are more likely to actively seek information before purchasing products claiming to have ecological properties [51].

Concerning Hypothesis 2a, it was confirmed that environmental concern positively predicts positive perceptions of circular products (products with recycled/reused content, as well as repaired or refurbished products). The confirmation of this hypothesis suggests that environmental concern could be fostered among the population through various awareness campaigns, thus increasing the frequency of the purchase of such products [52,53]. Moreover, the attitude towards circular products mediated the relationship between environmental concern and circular purchase behaviour (Hypothesis 2b).

Nevertheless, our findings shows that climate skepticism has a significant positive impact on perceived greenwashing (Hypothesis 3a), but a non-significant negative impact on the attitude towards circular products (Hypothesis 3b). This is in contrast to prior published papers that found significant relationships between climate skepticism and negative attitudes toward environmental products or initiatives [54,55]. The rejection of this hypothesis can be attributed to either the convenience sample or, from a theoretical standpoint, the relationship between the predictors and the criterion can be explained by other mediator variables that drive the total effect of these relationships.

Thus, in addition to the obvious practical implications of these findings for the efficacy of environmental campaigns, we believe that particularly Hypotheses 1b and 3a provide insights for stakeholders seeking to present their ecological products in a genuine manner, free of greenwashing claims. In this regard, they should be aware that intrinsic consumer factors such as environmental concern and climate skepticism may have an impact on the marketing effectiveness of their circular products, thus consumer trust can be one factor worth investing in. Therefore, in this context, understanding the internal and external drivers that lead to consumers’ acceptance of circular products is crucial for the eventual uptake of these products in the market.

### 5.2. Limitations and Further Research Directions

Although the results offer insightful information regarding the circular economy’s behavioral segment, they must also be viewed critically, keeping in mind several limitations. 

Firstly, the cross-sectional nature of this study prevents us from drawing any conclusions regarding the causal relations between the variables examined. Although we can demonstrate the associative and predictive nature of the relations, unlike in experimental or longitudinal designs, the conditions for causality are not met.

On the other hand, the selection of participants was not random, causing unrepresentativeness of certain socio-demographic characteristic (e.g., occupational status and income). Moreover, despite its advantages, administering the questionnaire online does not allow any kind of control over the respondent’s environment, making it difficult to communicate in the case of concerns, requesting additional details, or protecting against possible disturbing factors. Therefore, in order to better validate the current research findings, future studies could also use a longitudinal research method. As the focus of this study is Romanian consumers, future studies may replicate or create similar models in countries with different cultural and economical backgrounds in order to generalize the findings. For instance, a cross-cultural approach could be considered in order to test consumer understanding of demand for circular products and their underlying drivers, particularly in Western Europe and North America in comparison with Eastern European countries.

## 6. Conclusions

Better product design and increasing product utility are critical to the development of a circular economy. However, the greatest emphasis has been placed on improving material and energy efficiency, as well as recycling various types of waste. The product-related inner circles of the circular economy—reuse, repair, recycle, or refurbish—received less attention, and strategies for widespread adoption of these concepts are less mature [10,56]. Understanding customers and the individual level factors that predispose them to purchase products that come from these inner cycles is essential for making effective use of the circular economy’s inner cycles.

This is why the primary goal of this research was to examine the individual-level factors that have an influence over circular purchasing behavior. We tested a path model of purchasing circular products that took into account environmental concern, climate skepticism, and the attitudinal factor towards circular products. As we previously stated, climate skepticism and environmental concern are factors that should be taken into consideration when trying to understand the decision behind buying a circular product. Among the previous studies that investigated customer awareness and interest in the circular economy, this paper contributes to the literature by providing some new insights relevant to understanding consumer behavior. First, we note that the effect of environmental concern on circular purchase behavior is not entirely direct, but rather mediated by the consumer’s attitude. Second, this study adds to the existing literature on greenwashing by demonstrating that intrinsic factors such as environmental concern and climate skepticism, in addition to company reputation or social corporate responsibility initiatives [57], can influence consumers’ perceptions of this practice.

The way in which products specific to the circular economy—products with recycled content, repaired, refurbished, or even repaired—integrate from the consumer’s perspective in a context in which marketing must increasingly take into account sustainable development remains a challenging topic that needs to be researched further. According to previous studies [58], people generally support environmental causes, but are not willing to change their lifestyle, and “green” products can occasionally be perceived as unpleasant, inconvenient, or odd. This may be because the industry has historically concentrated on developing environmentally friendly products without taking into account the products that actually fulfill consumers’ needs [59].

## Figures and Tables

**Table 1:** Socio-demographic characteristics of the sample.

	Condition	%
Gender	Female	51.4%
	Male	48.6%
Age	18−25 years	37.8%
	26−30 years	7.6%
	31−40 years	18.6%
	41−50 years	20.5%
	51−60 years	10.6%
	60+ years	4.9%
Occupational status	Employee	54.9%
	Entrepreneur	11.7%
	Student	28.8%
	Unemployed	1.3%
	Other	3.5%
Education	Middle school	0.6%
	High school	29.8%
	Bachelor’s degree	24.8%
	Master’s degree	28.4%
	Doctoral degree	12.2%
	Post-doctoral studies	4.2%
Monthly Income	Under 200 €	22.4%
	201−400 €	5.8%
	401−600 €	12.1%
	601−800 €	15.5%
	801−1000 €	9.4%
	1000 € +	34.8%

**Table 2:** Measurement instruments and their reliability.

Variable	Scale Type	N of Items	Alpha Cronbach
Environmental Concern	Likert 1—Strongly Disagree, 5—Strongly Agree	4 items Examples: “Resource waste is a severe issue, and we are not doing enough to promote recycling of waste”, “I am concerned about the rising use of natural resources and how that will affect present and future generations.”	0.848
Climate Skepticism	Likert 1—Strongly Disagree, 5—Strongly Agree	11 items Examples: “Claims that human activities are changing the climate are exaggerated”, “I do not think climate change is a real problem.”	0.890
Perceived Greenwashing	Likert 1—Strongly Disagree, 5—Strongly Agree	5 items Examples: “Most companies use misleading messages about the ecological characteristics of their products”	0.907
Attitude towards Circular Products	Likert 1—Strongly Disagree, 5—Strongly Agree	8 items Examples: “Are products containing reused or recycled material or components, in your opinion, of higher quality than traditional products?” Do you believe that repaired/refurbished products have a longer life cycle?”	0.873
Circular Purchasing Behaviour	Likert 1—Rarely 5—Very Often	7 items Examples: “When I buy groceries, I try to choose products with multipurpose packaging (e.g., reusable jars)”, ”How often do you buy products that have been repaired?”	0.879

**Table 3:** Descriptive statistics.

Variable	Mean	SD	Skewness	Kurtosis
Environmental Concern	4.409	0.743	−0.983	1.562
Climate Skepticism	2.060	0.813	0.793	0.315
Perceived Green Washing	3.384	0.864	−0.408	−0.100
Attitude towards Circular Products	2.992	0.749	−0.058	0.261
Circular Purchasing behaviour	2.887	0.873	−0.194	−0.391

**Table 4:** Pearson correlations for all of the study variables.

Variable	1	2	3	4	5
1. Environmental Concern	1				
2. Climate Skepticism	−0.359 **	1			
3. Perceived Green Washing	0.123 **	0.144 **	1		
4. Attitude towards Circular Products	0.268 **	−0.048	0.054	1	
5. Purchasing behaviour	0.270 **	−0.100 *	0.050	0.396 *	1

** *p* < 0.01; * *p* < 0.05.

**Table 5:** Model fit assessment.

Goodness of Fit Indices	CMIN/DF	NFI	GFI	AGFI	CFI	TLI	RMSEA	SRMR
Recommended value	≤3	≥0.9	≥0.9	≥0.9	≥0.9	≥0.9	≤0.08	≤0.08
Proposed model	0.176	0.998	1.000	0.998	1.000	1.032	0.000	0.0067

**Table 6:** Results of hypothesis testing.

Hypothesis	Constructs	Standardized Estimate	Standard Error	Critical Ratio	*p*-Value	Decision
H1a	EC → CPB	0.176	0.046	4.475	0.00	Accepted
H1b	EC → PGW	0.201	0.51	4.586	0.00	Accepted
H2a	ACP → CPB	0.349	0.046	8.851	0.00	Accepted
H2b Mediation						
	EC → CPB	0.176	0.46	2.198	0.00	Accepted
	EC → ACP	0.288	0.044	6.636	0.00	
	ACP → CPB	0.349	0.046	8.851	0.00	
H3a	CS → PGW	0.216	0.047	4.942	0.00	Accepted
H3b	CS → ACP	0.055	0.040	1.267	0.20	Rejected

EC = environmental concern, CPB = circular purchase behaviour, GCP = green creative performance, PGW = perceived greenwashing, ACP = attitude towards circular products, CS = climate skepticism.

## Data Availability

Not applicable.
